# Computer vision syndrome among Sudanese medical students: a growing medical issue in the digital world

**DOI:** 10.1097/MS9.0000000000001917

**Published:** 2024-03-06

**Authors:** Mumen Abdalazim Dafallah, Omer Ali Mohamed Ahmed, Malaz Mustafa Ibrahim Mohamed, Rawan Abdalrahman Zakaria Abubakar, Ahmed Alsiddig Ebraheem, Gamal M. A. Ahmed

**Affiliations:** a Department of Internal Medicine, Sudan Medical Specialization Board; b Faculty of Medicine; Departments ofc Internal Medicine; d Surgery, Faculty of Medicine, University of Gezira, Wad Medani, Gezira State, Sudan

**Keywords:** computer vision syndrome, CVS, medical students, medical education, Sudan

## Abstract

Computer vision syndrome (CVS) refers to a set of eye-related symptoms that arise from prolonged computer usage. A survey was conducted to investigate the demographic characteristics, factors contributing to, and preventive measures against CVS. Out of 159 participants, 31.0% experienced seven or more symptoms, indicating a notable prevalence. The study found no significant correlation between age or academic years and CVS occurrence (*P* values of 0.481 and 0.392, respectively). However, gender exhibited a statistically significant relationship, with females students showing a higher prevalence than males (*P*=0.018; τ=0.105*). Notably, the distance from the screen had a highly significant inverse correlation with CVS occurrence (*P*=0.000; τ=−0.207**), indicating that greater distance reduced the risk. Additionally, using a screen filter (*P*=0.000; τ=0.184**) and adjusting screen brightness (*P*=0.017; τ=0.101*) were associated with CVS occurrence. Among preventive measures, only the use of an anti-glare screen showed a significant association with reducing CVS risk (*P*=0.018; τ=−0.099*). Given these findings, raising awareness about CVS among medical students is recommended, especially as curricula in medical colleges evolve.

## Introduction

Highlights

- The demographic characteristics, contributing factors and preventive measures associated with the development of computer vision syndrome (CVS) have been assessed.
- A significant number of medical students experienced CVS symptoms.
- Distance from the screen, screen brightness, and the use of screen filters were significantly associated with CVS.
- Medical students should be introduced to positive preventive coping strategies for example the use of anti-glare screens. Such interventions should be applied with a particular focus on those who used the smart devices constantly.
- Medical colleges should raise awareness among medical students about CVS and its harmful effects through educational programs. Since the Colleges of Medicine constantly develop their curriculum, raising awareness regarding CVS is advisable.

Computer vision syndrome (CVS), also known as digital eye strain (DES), refers to a cluster of eye symptoms brought about by prolonged utilization of computers or electronic devices^1^. The prevalence of CVS has risen in tandem with the widespread adoption of devices such as computers, laptops, smart phones, and tablets, prompting concerns about its potential as a public health issue^2^.

Although the exact pathophysiology remains incompletely understood, CVS development is commonly associated with three primary mechanisms: ocular surface, extraocular surface, and accommodative mechanisms. Ocular surface issues may manifest as dryness, redness, or a burning sensation, while extraocular surface problems can result in pain, headaches, and stiffness in the back, shoulders, and neck. Accommodative issues can cause blurred or double vision and difficulty with focus adjustment. With increasing time spent in front of digital screens, individuals of all ages and genders are susceptible to CVS, particularly when visual demands surpass an individual’s visual capabilities, leading to discomfort. According to the American Optometric Association (AOA), these symptoms are frequently associated with activities involving close vision^3–5^.

The AOA has categorized CVS symptoms into visual, ocular, and extraocular categories, encompassing classical symptoms like eye redness, itching, pain, blurred vision, headaches, and neck and shoulders discomfort^1,2^.

Prior research has linked CVS to various factors, including screen brightness, inadequate lighting, and the absence of anti-glare screens. Poor sitting posture, short distances from the screen, and screen positioning have also been identified as contributing factors^2,6–8^. The AOA has recommended preventive measures for computer users, such as following the 20-20-20 rule, ensuring frequent blinking, and maintaining sufficient room illumination^9–12^.

Given the extensive use of electronic devices among medical students, there’s a heightened risk of CVS development. A study in Sudan highlighted a high prevalence of CVS among medical students, with sitting posture correlating with symptom severity^9^. However, there’s a dearth of data on CVS within our context. Hence, our objective is to raise awareness of this condition among Sudanese medical students, who predominantly rely on electronic devices for studying rather than traditional textbooks.

## Results

A total of 513 medical students took part in the survey, with females comprising 61.6% and males 38.4%, resulting in a female-to-male ratio of 1.604:1. The participants’ mean age was 21 years (SD=2.2 years, range=17–27). The majority (71.4%) were under 20 years old, while 66.6% fell between 20 and 24 years, and 84.6% were aged 25 and above. Across academic years, the distribution was: first year (19.3%), second year (18.5%), third year (17.7%), fourth year (28.3%), and fifth year (16.2%). Among them, 159 participants (31.0%) reported experiencing seven or more symptoms, classifying them as having CVS (Table 1).

**Table 1:** The prevalence of computer vision syndrome among the study participants

Study participants	Negative (participants who does not CVS) frequency, *n* (%)	Positive (participants who have CVS) frequency, *n* (%)	Prevalence of CVS
	354 (69)	159 (31)	31%

CVS, computer vision syndrome.

Kendall’s rank correlation coefficient and two-tailed significance tests were employed to assess the relationship between demographic characteristics and CVS occurrence. Results indicated no statistically significant relationship between age or academic year and CVS occurrence (*P*=0.481 and 0.392, respectively). However, a significant association was observed between gender and CVS, with females showing a higher prevalence (*P*=0.018). The correlation was positive and significant at the 0.05 level (τ=0.105*) (Table 2).

**Table 2:** The relation between the demographical characteristics with the occurrence of computer vision syndrome among the study participants

Demographic data	Sub-item	Negative (participants who does not CVS) frequency, *n* (%)	Positive (participants who have CVS) frequency, *n* (%)	Correlation (Kendall’s Tau-b)	Significant (two-tailed) *P* value
Age (year)	<20	152 (71.4)	61 (28.6)	0.031	0.481
	20–24	191 (66.6)	96 (33.4)		
	25 and above	11 (84.6)	2 (15.4)		
Sex	Male	148 (75.1)	49 (24.9)	0.105*	0.018
	Female	206 (65.2)	110 (34.8)		
Academic year	First year	86 (86.9)	13 (13.1)	0.034	0.392
	Second year	55 (57.9)	40 (42.1)		
	Third year	53 (58.2)	38 (41.8)		
	Fourth year	96 (66.2)	49 (33.8)		
	Fifth year	64 (77.1)	19 (22.9)		

CVS, computer vision syndrome.

* Correlation is significant at the 0.05 level (two-tailed).

The most commonly reported symptoms were headaches (82.3%), worsened vision (68.0%), and excessive blinking (58.5%) (Table 3).

**Table 3:** The symptoms and distribution of the study participants according to the presence of computer vision syndrome symptoms

Symptoms	Frequency (percentage), *n* (%)
Headache	422 (82.3)
Feel that the sight is worsening	349 (68.0)
Excess blinking	300 (58.5)
Redness	258 (50.3)
Increase sensitivity to light	229 (44.6)
See coloured halos around objects	202 (39.4)
Dryness	199 (38.8)
Pain	190 (37.0)
Blurred vision	179 (34.9)
Itching	151 (29.4)
Excessive tearing	143 (27.9)
Burning sensation	141 (27.5)
Foreign body sensation	114 (22.2)
Difficulty focusing on near vision	102 (19.9)
Double vision	83 (16.2)
7 symptoms and more	159 (31.0)

Regarding factors associated with CVS, a majority of participants (44.8%) reported studying for 3–4 h daily, with 40.2% studying for over 4 h. Additionally, 91.2% reported taking study breaks, ranging from every 30 min or less (27.8%) to every 60 min or more (29.4%).

Kendall’s rank correlation coefficient and the Sig. (two2-tailed) tests were used to examine the relationship between the associated factors and the occurrence of CVS among participants. The results showed that the distance from the screen had a highly statistically significant relationship with the occurrence of CVS (*P*=0.000), and the correlation was inverse and significant at the 0.01 level (τ=−0.207**). The use of a screen filter also had a highly statistically significant relationship with the occurrence of CVS (*P*=0.000), and the correlation was direct and significant at the 0.01 level (τ=0.184**). The brightness of the screen also had a statistically significant relationship with the occurrence of CVS (*P*=0.017), and the correlation was direct and significant at the 0.05 level (τ=0.101*) (Table 4).

**Table 4:** The relation between the associated factors with the occurrence of computer vision syndrome among the study participants

Risk factors	Sub-item	Negative (participants who does not CVS) frequency, *n* (%)	Positive (participants who have CVS) frequency, *n* (%)	Correlation (Kendall’s Tau-b)	Significant (two-tailed) *P* value
Duration of the study	1–2 h/d	51 (66.2)	26 (33.8)	0.030	0.477
	3–4 h/d	167 (72.6)	63 (27.4)		
	More than 4 h/d	136 (66.0)	70 (34.0)		
Taking breaks	Yes	324 (69.2)	144 (30.8)	0.016	0.723
	No	30 (66.7)	15 (33.3)		
Distance from the screen	>Arm and forearm length	135 (58.4)	96 (41.6)	−0.207**	0.000
	< Arm and forearm length	219 (77.7)	63 (22.3)		
Posture	Sitting	107 (69.5)	47 (30.5)	0.005	0.907
	Lying	209 (68.8)	95 (31.3)		
	Both	38 (69.1)	17 (30.9)		
Level of the screen	Below level of the eyes	207 (70.6)	86 (29.4)	0.037	0.389
	Same level of the eyes	129 (66.5)	65 (33.5)		
	Above level of the eyes	18 (69.2)	8 (30.8)		
Source of lightening	From the ceiling/wall	244 (67.2)	119 (32.8)	−0.060	0.173
	Other sources	110 (73.3)	40 (26.75)		
Brightness of the screen	Very bright	73 (75.3)	24 (24.7)	0.101*	0.017
	Bright	224 (70.0)	96 (30.0)		
	Dull or dark	57 (59.4)	39 (40.6)		
Using screen filters/anti-glare screen	Yes	204 (77.3)	60 (22.7)	0.184**	0.000
	No	150 (60.2%)	99 (39.8%)		

CVS, computer vision syndrome.

* Correlation is significant at the 0.05 level (two-tailed).

** Correlation is significant at the 0.01 level (two-tailed).

Only 11.9% of participants reported always/very often adhering to the 20-20-20 rule, while 32.7% reported always/very often engaging in frequent blinking. Kendall’s rank correlation coefficient and the Sig. (two-tailed) tests were used to examine the relationship between preventive measures and the occurrence of CVS among participants. Importantly, the results showed that the use of an anti-glare screen was statistically significantly associated with a reduced likelihood of developing CVS (*P*=0.018), and the correlation was inverse and significant at the 0.05 level (τ=−0.099*). The other preventive measures were not associated with a reduced likelihood of developing CVS among study participants (Table 5).

**Table 5:** The relation between the preventive measures with the occurrence of computer vision syndrome among the study participants

Preventive measures	Sub-item	Negative (participants who does not CVS) frequency, *n* (%)	Positive (participants who have CVS) frequency, *n* (%)	Correlation (Kendall’s Tau-b)	Significant (two-tailed) *P* value
20-20-20 rule	Always/very often	159 (65.2)	85 (34.8)	−0.059	0.162
	Occasionally	154 (74.0)	54 (26.0)		
	Rarely/never	41 (67.2)	20 (32.8)		
Frequent blinking	Always/very often	73 (74.5)	25 (25.5)	−0.028	0.504
	Occasionally	156 (63.2)	91 (36.8)		
	Rarely/never	125 (74.4)	43 (25.6)		
Optimal location of the screen	Always/very often	90 (65.2)	48 (34.8)	−0.038	0.364
	Occasionally	155 (70.5)	65 (29.5)		
	Rarely/never	109 (70.3)	46 (29.7)		
Optimal lightening	Always/very often	67 (59.8)	45 (40.2)	−0.038	0.364
	Occasionally	148 (73.6)	53 (26.4)		
	Rarely/never	139 (69.5)	61 (30.5)		
Optimal sitting location	Always/very often	70 (75.3)	23 (24.7)	0.009	0.825
	Occasionally	156 (65.3)	83 (34.7)		
	Rarely/never	128 (70.7)	53 (29.3)		
Using of anti-glare screen	Always/very often	148 (63.8)	84 (36.2)	−0.099*	0.018
	Occasionally	99 (71.7)	39 (28.3)		
	Rarely/never	107 (74.8)	36 (25.2)		

CVS, computer vision syndrome.

* Correlation is significant at the 0.05 level (2-tailed).

## Discussion

A total of 159 participants reported experiencing a minimum of 7 symptoms during digital device usage, resulting in an overall prevalence of 31.0%. Compared to previous studies which reported a wide prevalence range of 12–97% among medical students, our study’s prevalence falls within the lower range^2^. Notably, our prevalence is lower than those reported in studies from China (74.3%), India (77.5%), Egypt (86.0%), and Sudan medical schools (94.5%)^9,12–14^, but higher than a study from Japan^12^. This difference may be attributed to variations in diagnostic methods and tools used to diagnose CVS. Previous studies often reported the prevalence of CVS as having one or more symptoms during computer use. Additionally, differences in geographical distribution, study period, socioeconomic status, and lack of knowledge regarding preventive measures of CVS could also contribute to these variations. The possibility of subjective responses in cross-sectional studies like ours may also influence prevalence estimates. Our prevalence is also lower than that of the general population (77.6%), computer workers (67.4%), and bank workers (73.0%)^15–17^.

Our study found a significant association between gender and CVS development, with females exhibiting a higher prevalence, consistent with several previous studies^16,18,19^. However, conflicting reports exist, with some studies reporting higher prevalence among males^20^. Possible explanations for this gender discrepancy include increased stress among females due to additional domestic and parental responsibilities and hormonal fluctuations, although further investigation is warranted^21^. Interestingly, we found no significant relationship between age, academic year, and CVS development, consistent with previous findings^19^. However, other studies have noted a significant association between CVS and age above 40 or 45 years^16,22^.

Regarding CVS-associated factors, our results highlighted the importance of screen distance and the use of screen filters, both significantly associated with CVS development. These findings align with previous research^19,23^, although the efficacy of screen filters in reducing CVS prevalence has been debated^19,24^.

Using a screen filter can reduce eye strain, enhance the focusing process, and improve visual comfort and productivity. The ideal distance from the screen depends on the type and size of the screen, but an arm’s length away from the screen (20–40 inches) is considered suitable for computer users according to the AOA^25,26^. This highlights that a shorter distance of less than 20 inches leads to a higher risk of developing CVS. Adjusting screen brightness was also associated with CVS, consistent with prior findings^19^. Contrary to some studies, we did not find a significant association between sitting position and CVS development. Bright lights often contribute to discomfort glare, so adjusting the light with a filter to an acceptable level is encouraged. This process will help reduce visual fatigue and the risk of developing CVS^3^.

The position of studying was not found to be associated with CVS in our study, which is in contrast to a study by Gadain *et al*. who concluded that sitting position was associated with the development of CVS among Sudanese medical students^9^. In our study, 47.8% of the respondents believed that they should take a break every 30–60 min, while 27.8% believed that they should take a break for 30 min or less. These responses varied from another study conducted in Saudi Arabia^27^. Previous studies support our finding that taking breaks was not significantly associated with CVS^16,24^. However, students should still be encouraged to take breaks when using electronic devices, as it can reduce the risk of developing other eye diseases. Interestingly, the duration of the study was also not found to be associated with CVS in our study, which contrasts with previous studies that reported a higher prevalence of symptoms with longer durations^24,26^.

Preventive measures play a crucial role in reducing CVS symptoms. These measures are supported and approved by the AOA to reduce the burden of CVS^26^. Some of these measures include the 20-20-20 rule, proper sitting position with adjustment of light and glare, and frequent blinking. Our study revealed that the use of anti-glare screens was significantly associated with reduced CVS prevalence, while other preventive measures did not show such association. The 20-20-20 rule helps reduce eye strain and fatigue by reminding individuals to look at an object 20 feet away for 20 sec every 20 min. Only 11.9% of our participants reported always or very often using this technique, which is similar to previous studies^9,28^. In contrast, the 20-20-20 rule has been found to reduce the risk of CVS in those studies^19,29^. The underutilization of strategies like the 20-20-20 rule and proper sitting posture among participants suggests a need for greater awareness and adherence to these measures.

The AOA recommends placing the screen at 10–20° below eye level. A higher angle may increase CVS symptoms by exposing more of the cornea and conjunctiva^26^. Straker *et al*.^30^ found that CVS symptoms worsen with sitting position, but our study did not find an association. Another study also found no significant association between sitting position and CVS^19^. Frequent blinking helps clean the eyes, provide oxygen and nutrients, and improve image clarity on the retina, keeping the eyes comfortable and healthy. However, one study from Portugal found that a decrease in blinking rate was directly associated with CVS^31^, which contrasts with our findings.

Despite the potential public health impact of CVS, particularly during the COVID-19 era with increased digital device usage, there remains a paucity of research, especially in developing countries. Abed Alah and colleagues conducted a study on CVS during remote learning and emphasized the importance of evidence-based strategies to prevent CVS. They also recommended conducting longitudinal studies to further understand CVS and its harmful effects^32^. Future longitudinal studies are warranted to better understand CVS and its preventive strategies.

Despite the potential for CVS to become a major public health concern, little is known about it, especially in developing countries^33^. Therefore, further studies and research need to be conducted.

### Strengths and limitations

Most surveys diagnose CVS based on the presence of one or more symptoms, which may overestimate the true prevalence of this condition. Our study used the Italian version, which requires the presence of greater than or equal to 7 symptoms for diagnosing CVS. This score is simple, valid, and reliable scale for the assessment and diagnosis of CVS in the adult digital-device-using population in all types of studies.

Since our study was cross-sectional, it did not allow us to study cause-effect relationships and is limited by subjective questions, which may introduce response bias. The response bias usually occurred when items are unclear or poorly structured, as a result, we are eager and vetted to provide easily and well-structured questionnaire that doesn’t underestimate the propensity of individuals to misunderstand the questions. Also, we aimed to focus on a specific events and experiences during use of the smart devices for a better response by the study participants.

By ensuring a common language between us and the participants, providing enough options in the answers and targeting the right participants (medical students); we aimed to reduce the response bias in our survey. In addition, we aimed to provide easy questions that minimize memory distortion, and conducting a pilot study to reduce the likelihood of the recall bias.

Assuring anonymity and confidentiality, along with clear instructions and communication about the purpose of the questionnaire were also achieved to avoid confusion, ensure understanding, and guarantee more accurate responses. Furthermore, the study had the strength of a large sample size and participants from different parts of the country.

## Conclusions

A notable proportion of medical students reported experiencing symptoms of CVS. Factors such as screen distance, brightness, and the utilization of screen filters were found to be significantly linked to the occurrence of CVS. Introducing proactive preventive measures, such as the adoption of anti-glare screens, is crucial, especially for individuals who frequently use smart devices. Medical colleges should prioritize raising awareness about CVS and its detrimental effects through educational initiatives, considering the evolving nature of medical curricula. Moreover, further prospective studies are needed to better understand causal relationships and risk factors associated with CVS.

## Methods

### Settings and participants

This descriptive cross-sectional community-based study was carried out from 15 January to 22 February 2023, at one of Sudan’s largest medical colleges. The study encompassed medical students originating from various states within Sudan, as well as those from outside the country. This diverse participant pool facilitated the representation of a broad spectrum of academic and cultural backgrounds.

### Sample size

The target participants for the academic year numbered approximately 1747, distributed across six batches, two of which were in the fourth year. A questionnaire was randomly distributed among medical students to gather data, resulting in a total of 513 responses. This sample size was calculated using the online Rao soft sample size calculator http://www.raosoft.com/samplesize.html, considering a total population of 1747, a 99% confidence interval, a 5% margin of error, and a 50% response distribution. The minimum required sample size was determined to be 483, but the study surpassed this with 513 participants.

### Study design and questionnaire

All study participants were briefed on the study objectives before their involvement. Content validation was undertaken by a panel of experts to ensure the accuracy and representativeness of the data. Additionally, face validation was conducted to ascertain the questionnaire’s effectiveness and alignment with the study’s goals. The validation and cross-cultural adaptation process for our research questionnaire involved bilingual experts from both the medical education and ophthalmology departments in our college. A pilot test evaluated the clarity and cultural relevance of the translated Italian version of the questionnaire. Subsequently, all authors reviewed the feedback, made necessary adjustments, and produced a pre-final version. This version was back-translated to ensure data accuracy. Another round of pilot testing was conducted on a separate group meeting the same inclusion criteria, leading to the establishment of the final version by consensus among all authors and the medical educationalist. Finally, the ethical committee was consulted to ensure the questionnaire’s validity and reliability. The questionnaire comprised four sections: demographic data, ocular and extraocular symptoms, predisposing factors, and preventive measures adopted by the medical students.

### Data management and analysis

Frequency and percentage were computed for demographic data, symptoms, risk factors, and preventive measures using SPSS version 26. Mean, standard deviation, and interquartile range were calculated for age. The relationship between demographic characteristics, associated factors, preventive measures, and the occurrence of CVS was analyzed using bivariate analysis, specifically the non-parametric Kendall’s rank correlation coefficient and the Sig. (two-tailed) test.

The non-parametric Kendall’s rank correlation coefficient assesses the strength and direction of association between the variables under examination. A smaller coefficient indicates a higher number of inversions. The Sig. (two-tailed) test, interpreted using the *P* value, determines the statistical significance of the association. If the *P* value is less than 0.05 or 0.01, it signifies a statistically significant association between the variables.

The symbol * denotes a significant correlation at the 0.05 level (2-tailed), while ** indicates a significant correlation at the 0.01 level (two-tailed). Therefore, the analysis initially employed the non-parametric Kendall’s rank correlation coefficient to describe the relationship between variables, followed by the Sig. (two-tailed) test to ascertain its statistical significance.

### Symptoms and scale calculator

Most of the questions in our questionnaire were adapted from previous studies^34,35^, with adjustments made to ensure relevance to Sudanese culture and society. This adapted questionnaire underwent validation and reliability testing through a small pilot study. Participants were asked to indicate whether they experienced specific symptoms during the use of their computer devices. We included 15 symptoms associated with computer vision syndrome (CVS). A score of greater than or equal to 7 on these symptoms indicates the presence of CVS^2,3^. Our questionnaire was based on a validated Italian version, known for its simplicity, validity, and reliability in assessing and diagnosing CVS in adult digital-device users across various study settings.

### Ethics approval and consent to participate

All procedures in this study adhered to ethical guidelines, with participants thoroughly briefed on the study objectives. The authors verbally communicated the study’s objectives to all participating students. Subsequently, paper-based questionnaires were distributed to collect data from participants who provided verbal consent. Written consent was obtained through participants reading the covering letter in the questionnaire and expressing agreement by completing the survey after receiving verbal explanation of the study. Hence, both verbal and written consent were secured from participants. Confidentiality was rigorously maintained throughout all stages of the study. Ethical approval for this study was obtained from the Research Ethics Committee on 12 December 2022. Since this is solely an observational study without involvement of original human data from patients or hospital records, no registration number was required from the chairman of the Research Ethics Committee. Nevertheless, both the chairman and the research team reviewed and approved the study. This study adheres to the STROCSS criteria for reporting research work^36^.

## Ethical approval

Ethical approval for this study (Ethical Committee N° NAC 1-36) was provided by the Ethical Committee NAC of University of Gezira- Faculty of Medicine, Wad Medani, Sudan on 12 December 2022.

## Consent

Written informed consent was obtained from the patient for publication and any accompanying images.

## Source of funding

Not applicable.

## Author contribution

M.A.D., O.A.M.A. design, conceptualized the data. M.M.I.M. and R.A.Z.A. collect and analyze the data. A.A.E. and G.M.A.A. revised the paper critically. All authors agree to and approved the final publication of this article.

## Conflicts of interest disclosure

The authors declare no competing interest.

## Research registration unique identifying number (UIN)

Not applicable.

## Guarantor

Mumen Abdalazim Dafallah.

## Data availability statement

The datasets used and/or analyzed during the study are available from the corresponding author on reasonable request.

## Provenance and peer review

NA.
